# Polyphenols contribute to the antioxidant and antiproliferative activity of *Phyllanthus debilis* plant *in-vitro*

**DOI:** 10.1186/s12906-016-1324-5

**Published:** 2016-09-01

**Authors:** Dananjaya Perera, Preethi Soysa, Sumedha Wijeratne

**Affiliations:** 1 Department of Biochemistry and Molecular Biology, University of Colombo, Faculty of Medicine, Colombo 08, Sri Lanka; 2 Department of Obstetrics & Gynaecology, University of Colombo, Faculty of Medicine, Colombo 08, Sri Lanka

**Keywords:** Phyllanthus debilis, Polyphenols, Antioxidant activity, Anti-proliferative activity, RD, CC1

## Abstract

**Background:**

*Phyllanthus debilis* (Elapitawakka) is a medicinal plant used in traditional systems of medicine in Sri Lanka. Present study was carried out to evaluate in-vitro anti-oxidant and anti-proliferative activity of the water extracts of aerial parts (AP) and roots (RP) of *P*.*debilis* plant and the role of polyphenolic compounds in view of its medicinal use.

**Method:**

Total polyphenols, flavonoids and proanthocyanidin content of the extracts were quantified. DPPH, hydroxyl radical, nitric oxide and hydrogen peroxide scavenging potentials and the total antioxidant capacity, ferric ion reducing power were determined to evaluate antioxidant capacity. Anti-proliferative activity was assessed with MTT assay for Human Rhabdomyosarcoma (RD) and normal rat liver cells (CC1) after 24 h exposure to the plant extracts. DPPH and MTT assays were carried out for AP and RP extracts after removal of polyphenols to assess the contribution of polyphenols on antioxidant and anti-proliferative activity of *Phyllanthus debilis*.

**Results:**

Flavonoid content of the AP extract was significantly lower than that of RP (*P* < 0.001) while no significant difference was observed in polyphenolic as well as in proanthocyanidin contents. All the assays except for phosphomolybdate assay demonstrated that the RP extract had higher antioxidant capacity (*p* < 0.001) compared to AP. Further, antioxidant capacity and anti-proliferative activity were lower (*p* < 0.001) in AP and RP in the absence of polyphenols compared to the crude extract.

**Conclusion:**

Root contains higher levels of flavonoids than the aerial part. Moreover, the presence of polyphenols is required for antioxidant and anti-proliferative activities of both AP and RP.

## Background

Reactive free radicals are constantly generated in human body through biological reactions. Reactive oxygen species (ROS) such as hydroxyl radical (OH^.^), hydrogen peroxide (H_2_O_2_), superoxide radical (O_2_) and singlet oxygen (O_2_^.^) and reactive nitrogen species (RNS) such as nitric oxide (NO˙), peroxynitrite (ONOO^−^) and nitrogen dioxide (NO_2_) are the main sources of reactive free radicles. The production of free radicals are neutralized by endogenous antioxidants and enzymes, however excessive production of reactive molecules causes oxidative stress. As a result they oxidize biologically important macro molecules such as DNA, proteins, lipids and carbohydrates [1]. DNA strand breaks and abnormal DNA linkages caused by ROS and RNS are closely associated with carcinogenesis [2]. The consequence of oxidative stress is also associated with cardiovascular, metabolic, inflammatory diseases and various neurodegenerative diseases including Alzheimer’s disease, Parkinson’s disease and multiple sclerosis [3].

Plants produce broad spectrum of polyphenolic compounds as secondary metabolites for their survival under stress conditions. Polyphenols have an ability to neutralize free radicals by donating them electrons or hydrogen atoms which may play a vital role in health benefits [4].

*Phyllanthus debilis* (Elapitawakka/Bim nelli) is an annual plant found in Sri Lanka. The genus *Phyllanthus* (Euphorbiaceae) contains about 800 species in worldwide and most of these plants have been used as drugs in traditional systems of medicine all over the world [5]. Studies carried out on *P.debilis* with authenticated plant are very limited up-to date. Aqueous extract of *P.debilis* has shown anti-hyperglycemic and hypoglycemic activity in mice [6]. Further analgesic and anti-inflammatory activity have been demonstrated for the petroleum ether extract of the whole plant in animal models [7]. Anticancer, antibacterial and antioxidant activity have also reported with different solvent extracts in vitro [5, 8–10].

The whole plant of *P.debilis* or its parts are used to prepare porridge or ‘decoction’ in traditional medicine and folk medicine to treat ailments such as liver complications, diabetes mellitus and skin diseases in Sri Lanka [11]. The present study was carried out to explore the antioxidant activity and the potential anti-proliferative activity of the water extract of *P.debilis* plant as used in traditional systems of medicine. Since it is important to understand the contribution of polyphenols on antioxidant and anti-proliferative activity, further investigations were carried out with the plant extracts after removing their polyphenols.

## Methods

### Chemicals and equipment

Gallic acid, 2-deoxy-D-ribose, Folin-Ciocalteu’s reagent and other chemicals needed for cell culture and cell viability studies were purchased from Sigma Chemicals Co. (P.O. Box 14508, St. Louis, MO 63178 USA). 1,1-Diphenyl-2-picrylhydrazyl (DPPH) free radical, epigallocatechin gallate (EGCG), aluminium chloride, polyvinyl-polypyrrolidone (PVPP) were purchased from Fluka (Flukachemie GmbH, CH-9471 Buchs). L-Ascorbic acid, hydrogen peroxide, N-(1-naphthyl)-ethylene diaminedihydro chloride and ethanol were purchased from BDH Chemicals (BDH Chemicals Ltd, Poole, England). All chemicals used were of analytical grade.

SHIMADZU UV 1601 UV/Visible spectrophotometer (Shimadzu Corporation, Kyoto, Japan) was used to read the absorbance. LFT 600 EC freeze dryer was used to freeze-dry the plant extracts (LFT 600 EC, −90–95 °C temperature, 10 valves with Hitachi pump). Cells used for the assessment of anti-proliferative activity (RD, CC1) were incubated at 37 °C in a humidified CO_2_ incubator (SHEL LAB/Sheldon manufacturing Inc., Cornelius, OR 97113, USA). Olympus (1X70-S1F2) inverted fluorescence microscope (Olympus Optical Co. Ltd. Japan) and digital camera (MDC 200 (USB 2.0) 2 M pixels with CCD chip) were used for assessment and imaging of cell morphology.

RD cell line was kindly donated by Dr Sunethra Gunasena, Medical Research Institute, Colombo 08, Sri Lanka and CC1 (Normal rat liver fibroblast) cell line was obtained from Dr. Panjwani Center for Molecular Medicine and Drug Research, University of Karachi, Pakistan.

### Plant materials

*Phyllanthus debilis* plants in the seeding stage were collected from Pannipitiya area in the Colombo district, Sri Lanka (June 2014). The plant was taxonomically identified and voucher specimens were deposited in Botany Department, Bandaranayaka Memorial Aurveda Research Institute, Nawinna, Colombo, Sri Lanka (Deposition number: 755/a).

### Preparation of the decoctions

The plant materials were washed in distilled water followed by deionized water and air dried. The aerial parts and the root parts were separated and freeze dried until a constant weight was obtained. Dried samples were ground using a kitchen blender. The powdered aerial parts (50 g) were refluxed with 500 mL of deionized water for 3 h in triplicate. The roots were pooled together and the powdered roots (50 g) were refluxed as previously. Decoctions were filtered through a glass funnel plugged with cotton wool, then through a Whatman filter paper. Filtrate was centrifuged at 10,000 rpm for 15 min and the supernatant was freeze dried. The freeze dried samples were weighed and stored at −20 °C in sterile glass bottles until further use. The yield was calculated as a percentage of the dried plant material.

### Removal of polyphenols

Polyphenols were removed using Polyvinyl polypyrrolidone (PVVP) column as described by Soysa (1997) [12, 13]. Briefly, a cotton wool plug was placed inside a 5 cm^3^ syringe after removing the plunger and the needle. Syringe was filled with PVPP (1.7 g). Water extract of AP and RP (3 mL) was layered over the PVPP column. The PVPP column was placed in a 15 mL falcon tube and centrifuged at 2,000 g for 10 min. Centrifugation was repeated for 6 times with the same column adding 1 mL of the extract and each fraction was collected to separate tubes. The first fraction was discarded and remaining fractions were analyzed for the presence polyphenols. The Absorbance of AP (aerial parts) and RP (root parts) before and after PVPP treatment were scanned for wavelengths using a UV/Visible scanning spectrophotometer.

### Determination of total polyphenolic and flavonoid content

Total polyphenolic content (TPC) of the AP and RP were determined using Foiln-Ciocalteu’s method [14]. Gallic acid was used to construct the standard curve and total phenolic content was expressed as w/w% gallic acid equivalents (GAE w/w %).

The flavonoid content of the extracts was determined by aluminum chloride colorimetric assay [15]. Calibration curve was plotted using EGCG (− (−) -Epigallocatechingallate) standard and flavonoid content was expressed as w/w% EGCG equivalents (EGCGE w/w %).

### Determination of total proanthocyanidins

A volume of 2 ml of vanillin reagent (1 g vanillin in 50 mL, 70 % H_2_SO_4_) was mixed with 200 μL of the sample (400, 500, 700 μg/mL). The resulting mixture was allowed to stand for 15 min at room temperature. The absorbance was determined at 500 nm [16]. Calibration curve was constructed using EGCG and the total proanthocyanidin content was expressed as w/w % EGCG equivalent.

### Qualitative analysis of plant phytochemicals

Qualitative analysis for the phytochemicals were carried out using the methods described by Saeed et al., (2012) for alkaloids, tannins, terpenes, saponins, cardiac glycosides present in the water extracts of the plant [17].

### Phosphomolybdate assay for total antioxidant capacity

Total antioxidant capacity was determined using phosphomolybdate method [18]. Results were expressed as w/w% ascorbic acid equivalent (AAE).

### 2,2′- Diphenyl-1-picrylhydrazyl (DPPH) radical scavenging activity assay

In vitro antioxidant activity of the AP, RP, PFAP and PFRP were determined using stable free radical 2,2′- diphenyl-1-picrylhydrazyl (DPPH) [15]. Results were expressed as percentage inhibition calculated from the formula (1).

1 $$ \%\  Scavenge\  of\  DPPH\  free\  radical=\frac{\left( Ab\  of\  control- Ab\  of\  sample\right)\ X\ 100}{Ab\  of\  control} $$

### Hydroxyl radical scavenging assay

Hydroxyl radical scavenging activity of AP and RP was determined in lyophilized plant extracts. Fenton reaction was used to generate hydroxyl radicals in vitro [19]. The assay is based on quantification of degradation product of 2-deoxyribose by condensation with TBA. The percentage scavenging activity of hydroxyl radical was calculated using the formula (1). Ascorbic acid served as the positive control.

### Hydrogen peroxide scavenging activity

Assay was carried out according to the method developed by Fernando and Soysa [20]. Percentage scavenging activity was calculated using the formula (1). Ascorbic acid served as the positive control.

### Ferric ion reducing power

The reducing ability to convert ferric ions to ferrous ions by AP and RP was carried out as previously described [15]. Ascorbic acid served as the positive control.

### Nitric oxide radical scavenging

This assay is based on Griess Illosvoy reaction [15]. The pink azo-dye generated during reaction was measured at 540 nm. Ascorbic acid served as the positive control.

### Antiproliferative activity

#### Cell culture

RD (Rhabdomyosarcoma) and CC1 (normal rat liver) cells were cultured in tissue culture flasks with DMEM (Dulbecco’s Modified Eagle’s Medium) supplemented with 10 % FBS, 3 % glutamine, 1%penicillin-streptomycin, and 1 M HEPES (4-(2-Hydroxyethyl) piperazine-1-ethanesulfonic acid) buffer in humidified CO_2_ incubator in 37 °C.

### Cell morphology

Cell morphology was observed before and after the treatment of the extract at different concentrations using an inverted fluorescence microscope. Images of the cells were captured.

### MTT ((3-(4,5-Dimethylthiazol-2-yl)-2,5-diphenyltetrazoliumbromide)) assay

Cells were plated in 24-well flat bottom tissue culture plates at a density of 2 × 10^5^ cells/well and kept overnight at 37 °C in a CO_2_ incubator to obtain a 70 % confluent mono layer. The cells were treated with AP, RP, PFAP and PFRP at different concentrations for 24 h. Cells for negative controls were exposed to the same conditions without the plant extract. Cycloheximide (0.1 %, 50 μl) served as the positive control. Medium was replaced by MEM (1 ml) and MTT reagent (100 μl, 5 mg/ml). The contents were incubated for 4 h at 37 °C. Isopropyl alcohol (750 μl) in 0.05 M HCl was added to dissolve MTT crystals. Absorbance was recorded at 570 nm. Percentage cell viability was calculated using the formula 2 [21].

2 $$ \%\  Cytotoxicity = \frac{\left( Ab\  of\  Negative\  control- Ab\  of\  sample\right)\ X\ 100}{Ab\  of\  Negative\  control} $$

### Statistics and calculations

Each experiment was performed at least in triplicate for three lyophilized samples prepared from aerial extracts and for the pooled sample (lyophilized) of the root extract. Calibration curves were considered as linear if R^2^ > 0.99. The EC_50_ values were calculated from either linear or logarithmic dose response curves where R^2^ > 0.95 was considered as linear. Student *t* test was carried out for the statistical calculations using Microsoft Excel.

## Results and discussion

### Extraction yield, total phenolic, flavonoid and proanthocyanidin content

The extraction efficiency of aerial parts and the pooled sample of the roots were 12.1 (±0.5) and 12.5 % of the dry weight respectively. Total polyphenol content, flavonoid and proanthocyanidin content of extracts are depicted in Table 1. Mean values for TPC, flavonoid and proanthocyanidin content are higher in roots. However a significant difference was observed only with flavonoid content which is a subgroup of polyphenols. Significant difference was not observed for both polyphenolic and proanthocyanidin content between AP and RP (Table 1).

**Table 1:** Extraction yield, polyphenolic, flavonoid and proanthocyanidincontent of extracts

Part of the plant	Total polyphenolic content (w/w% GAE)	Flavonoid (w/w% EGCGE)	Proanthocyanidin (w/w% EGCGE)
Aerial parts (*n* = 3)	12.0 ± 0.6	65.9 ± 3.6*	13.8 ± 2.2
Roots (*n* = 3)	17.8 ± 3.7	82.6 ± 4.6*	14.5 ± 3.0

Results are expressed as mean ± SD. GAE -Gallic acid equivalent; EGCGE- (−) Epigallocatechingallate equivalent).*P < 0.001 when compared AP with RP. AP: aerial parts, RP: roots

Plant polyphenols are secondary metabolites, which synthesize for their defense against abiotic and biotic stress conditions. They also play numerous roles in biological systems such as antioxidants, antimicrobial and anticancer agents [22]. Gallic acid, rutin, corilagin, furosin and geraniin are some of the polyphenols characterized in aerial parts of *P.debilis* [10]. Flavonoids are also a subset of polyphenols. They involve in regulatory mechanisms in cell proliferation and differentiation to protect eukaryotic cells from oxidative stress through regulating the activity of different protein kinases [23]. Flavonoids have ability to scavenge free radicals and also serve to chelate metals that generate free hydroxyl radicals via the Fenton reaction [24]. The highest amount of flavonoid content was observed in RP than AP (*P* < 0.001) (Table 1). Proanthocyanidins is a subset of flavonoid which is synthesized in flavonoid biosynthetic pathway. They are oligomers of flavan-3-ol units with diverse biological activities [25]. Both roots and aerial part showed similar proanthocyanidin content. The present study also showed the presence of alkaloids, tannins, terpenes and saponins but the absence of cardiac glycosides. However a previous study reported that absence of saponin, triterpenoids, and tannins in *p.deblis* [26].

### Ferric ion reducing power

**Fig. 1:**
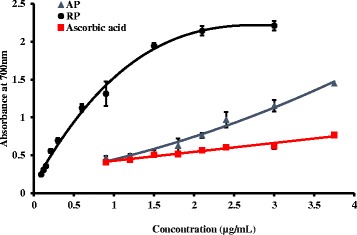
Ferric ion reducing capacity of plant extracts and L-Ascorbic acid standard at different concentrations. The results are presented as mean ± SD for shoot samples (*n* =3), root samples (*n* = 3) and L-Ascorbic acid (*n* =3)

Reducing potential is a marker of antioxidant capacity and it was assessed by ferric ion reducing assay. Ferric ions can be reduced to ferrous ions by the antioxidants present in the plant extracts. Ferric ion formation is monitored through the blue green colored complex at 700 nm [27]. Both parts of the plant showed higher reducing ability than the ascorbic acid standard. The capacity to reduce Fe ^3+^ by the root extracts is significantly higher (*p* < 0.05) than the ascorbic acid or AP at all the concentrations studied. Furthermore the root showed a reducing capacity even at 0.2 μg/mL (Fig. 1).

### Total antioxidant capacity

**Fig. 2:**
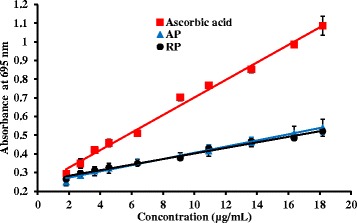
Total antioxidant capacity of plant extracts and L-Ascorbic acid at different concentrations. The results are presented as mean ± SD for AP (Aerial parts) (*n* =3), root samples (RP) (*n* = 3) and L-Ascorbic acid (*n* =3)

The assay is based on the reduction of molybdenum (VI) to molybdenum (V) which forms a green chromophore with phosphate in the acidic medium [28]. Total antioxidant capacity of both AP and RP depicts no significance difference (Table 2 and Fig. 2). Ferric ion reducing power results indicate higher reducing potential of root but molybdenum reduction assay demonstrates no significant difference in antioxidant potential in both extracts. This showed that though the capacity to reduce Fe^3+^to Fe^2+^is higher in roots, overlapping curves signifies (Fig. 2) that the capacity to reduce Mo^6+^ to Mo^5+^is similar in both parts. The positive control, ascorbic acid also showed different behavior in reducing power between ferric ion and molybdenum (VI) ion.

**Table 2:** EC_50_ values for free radical scavenging assays for hydroxyl, nitric oxide assay and hydrogen peroxide with relevant positive standards

EC _50_ values	OH^.^ (μg/mL)	NO^.^ (μg/mL)	H_2_O_2_ (μg/mL)	Total antioxidant capacity (w/w % AAE)
AP(Aerial parts) (*n* = 3)	49.27 ± 4.75*	211.05 ± 7.61*	42.54 ± 2.23*	37.7 ± 3.2
RP (Root) (*n* = 3)	27.77 ± 3.26*	170.75 ± 3.16*	30.23 ± 3.35*	35.3 ± 5.6
Gallic acid (*n* = 3)	27.37 ± 1.89	N/A	N/A	N/A
Ascorbic acid (*n* = 3)	N/A	212.58 ± 2.87	13.82 ± 1.11	N/A

Significant difference at *P < 0.001 when compared RP with AP. AAE: Ascorbic acid equivalent, N/A: Not applicable

### Hydroxyl radical scavenging assay

**Fig. 3:**
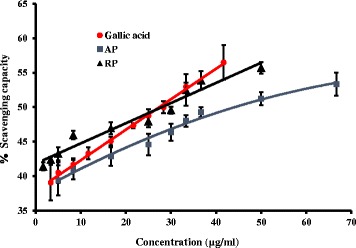
Dose response curve for percentage hydroxyl radical scavenging of plant extracts and L-Ascorbic acid standard. The results are presented as mean ± SD for aerial parts (AP), root parts (RP) and gallic acid standard

Hydroxyl radicals are generated by reducing the oxygen molecule to water and it is one of the main sources of reactive oxygen species in biological systems [29]. The values obtained for EC_50_ show that hydroxyl radical scavenging ability of RP is significantly higher than AP and no significant difference were found between root and ascorbic acid (Table 2). Further it was observed that the percentage scavenging ability of RP is higher at lower concentrations (below around 20 μg/mL) than the gallic acid. Scavenging capacity of AP is parallel to that of the gallic acid standard (Fig. 3).

### Nitric oxide radical scavenging assay

**Fig. 4:**
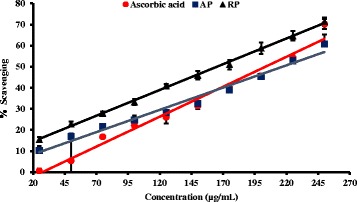
Dose response curve of nitric oxide radical scavenging percentage of plant extracts and L-Ascorbic acid standard. The results are presented as mean ± SD for AP (*n* =3), root samples (PR) (*n* = 3) and Gallic acid standard (*n* =3) AP: Aerial parts, RP: Root parts

Figure 4 Nitric oxide (NO) is a free radical and a signaling molecule which is responsible for various biological functions. It has been found that the peroxynitrite derived from NO under oxidative conditions is a highly reactive radical which can damage macromolecules [30]. The Present study showed that roots of *P.debilis* has high NO scavenging capacity and the EC_50_ is lower than the ascorbic acid (*p* < 0.001) (Table 2). These observations suggest that the possibility of using *P.debilis* in the treatment of degenerative diseases by scavenging nitric oxide free radicals. However further research should be carried out in this regard.

### Hydrogen peroxide scavenging assay (Fig. 5)

**Fig. 5:**
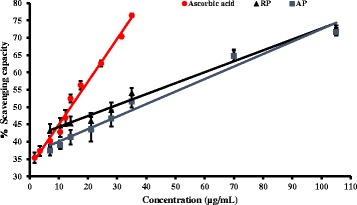
Dose response curve of percentage hydrogen peroxide scavenging ability of plant extracts and L-Ascorbic acid standard. The results are presented as mean ± SD for shoot samples (*n* =3), root samples (*n* = 3) and L-Ascorbic acid standard (*n* =3)

Hydrogen peroxide is a metabolic byproduct results from the action of superoxide dismutase with superoxide radical. Superoxide radicals have a capability of generating reactive oxygen species [31]. Hydrogen peroxide is less toxic than superoxide radical in biological systems. Both roots and aerial parts of the plants showed moderate activity against H_2_O_2_ scavenging activity. However the EC _50_ values were higher than the ascorbic acid reference standard (Table 2).

### Spectrum analysis

**Fig. 6:**
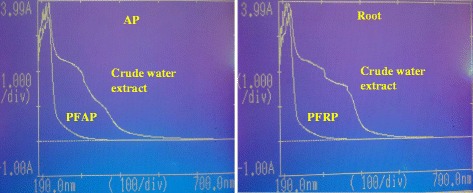
The photometric scanning spectrum obtained for the root and shoot samples before and after the treatment with PVPP. AP: Aerial parts, PFAP: polyphenol free aerial parts, PFRP: Polyphenol free root parts

The presence of polyphenols in both shoot and roots is evidenced by showing an absorbance peak between 190 and 700 nm. The broad peak observed around 235 nm was remained constant while the signals between the 230 to 500 nm were disappeared from both parts of the plant after PVPP treatment (Fig. 6). Data published by Shela et al., (1993) states that polyphenolic compounds in white wine had a maximum absorbance between 250 – 350 nm [32]. Similar observations were also found in the present study.

### Scavenging capacity for DPPH radical in the presence and absence of polyphenolic compounds of the plant extracts

**Fig. 7:**
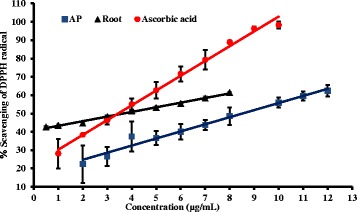
The dose dependent response curves for percentage scavenging of DPPH by water extracts of root and aerial parts (AP) of *P.debilis* plant comparison with L-Ascorbic acid. The results are presented as mean ± SD for shoot (*n* =3), root (*n* = 3) and L-Ascorbic acid (*n* =3)

Antioxidants readily react with DPPH radical change its original purple color into yellow [15]. The DPPH radical scavenging capacity of the root is comparable to ascorbic acid and significantly higher (*P* < 0.001) than the aerial parts (Table 3). Interestingly, DPPH scavenging capacity of the root extract at lower concentrations is higher than that of ascorbic acid but lower at high concentrations (Fig. 7). A previous study has reported that methanolic extracts of *P.debilis* has high DPPH scavenging activity and the results are comparable with the present study [33].

**Table 3:** DPPH scavenging assay, EC_50_ values with the presence and absence of polyphenols

Plant extract	EC_50_ values before removal of polyphenols (μg/mL)	EC_50_ values of after removal of polyphenols (μg/mL)
AP(Aerial parts) (*n* = 3)	8.6 ± 0.4	695.1 ± 4.6
RP (Roots) (*n* = 3)	3.7 ± 0.1	583.3 ± 12.3
Ascorbic acid (*n* = 3)	3.3 ± 0.3	-

**Fig. 8:**
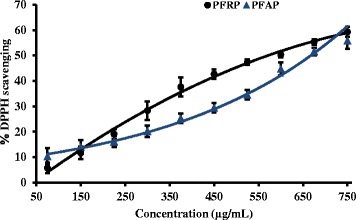
The dose dependent percentage scavenging potential of DPPH in the absence of polyphenols in AP and RP (*n* = 3). PFAP: Polyphenol free aerial parts, PFRP: Polyphenol free root parts. The results are presented as mean ± SD for AP

Polyvinyl polypyrrolidone (PVPP) is highly cross linked polymer which has high affinity towards polyphenols [13, 34]. In the absence of polyphenols both root and aerial parts showed high EC_50_ values for the DPPH assay. The EC_50_ ratios with the absence and presence of polyphenols are over 100 fold for both extracts. These results indicate that polyphenols are the main contributory factor for DPPH radical scavenging activity and involvement of non-polyphenols for the antioxidant activity is negligible. Spectrophotometric analysis suggests that the substance(s) which have maximum absorbance around 235 nm are not responsible for scavenging of DPPH (Fig. 8 and Table 3).

### Cell morphology

#### Anti-proliferative activity of AP and RP against RD and CC1 cells

**Fig. 9:**
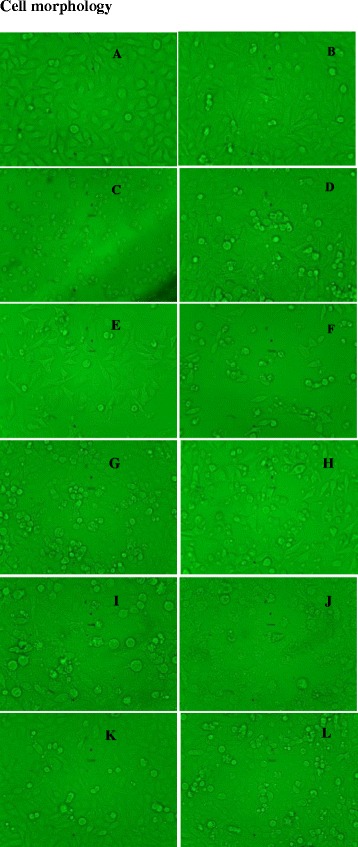
Morphological changes of cells observed after 24 h exposure of AP and RP extracts and cycloheximide (positive control) under light microscopy. **a** and **b** are negative controls of RD and CC1 cells. **c** and **d** are positive controls of RD and CC1 cells treated with cycloheximide (0.1 %, 50 μl) as the positive control respectively. **e** & **f**: RD Cells treated with AP at concentrations 200 and 500 μg/mL, respectively. **g** & **h**: CC1 Cells treated with AP at concentrations 800 and 1000 μg/mL, respectively. **i** & **j** RD cells treated with RP at concentrations at 200 and 500 μg/mL. **k** & **l**: CC1 cells treated with RP at concentrations 800 and 1000 μg/mL, respectively (20X)

Figure 9 depicts the morphological changes of RD cells treated with AP and RP extracts. Shrunken cells with condensed cytoplasm and membrane blebbing characteristics to apoptosis were observed in RD cells treated with plant extracts [35]. The cell death was concentration dependent for both AP and RP with the cell lines investigated. Normal rat liver fibroblast cells(CC1), showed apoptotic features characteristic to apoptosis only at high concentrations.

**Fig. 10:**
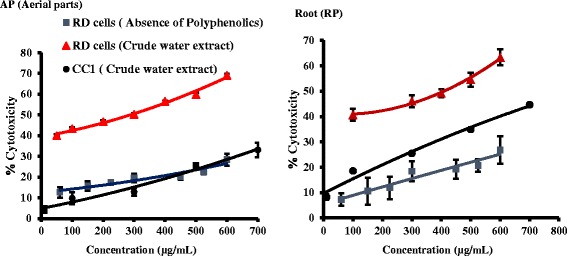
Dose dependent cytotoxic effect of AP and RP with RD and CC1 cells and cytotoxicity of polyphenol absence samples with RD cells, after 24 h exposure

Anti-proliferative activity was high in RD cells by both AP and RP extracts compared to the CC1 cells (Table 4 & Fig. 10). Even at a concentration such as 1200 μg/mL, the percentage cytotoxicity of CC1 cells was only 58.99 ± 2.43 % and 54.11 ± 0.88 % for AP and RP extracts respectively. Several compounds which have been isolated from *P.deblis* provide evidence for antioxidant and anti-proliferative activity confirmed in the present study. Gallic acid, rutin, corilagin, furosin and geraniin isolated from *P. deblis* and geraniin have shown high DPPH scavenging activity [10, 36]. It is reported that geraniin has antioxidant properties and induces apoptotic cell death in human lung adenocarcinoma cells in vitro and in vivo [37]. Gallic acid is a common naturally occurring phenol found inplants used for therapeutic purposes including *phyllanthus* species [38]. It acts as an antioxidant and exerts anti-proliferative effect on glioma T98G cells via dose-dependent epigenetic regulation mediated by miRNAs [39, 40]. The authors have further reported that the anticancer activity is based on its antioxidant effects.

**Table 4:** Comparison of EC_50_ values of the MTT cytotoxicity assay

	AP (*n* = 3)	RP (*n* = 3)
RD cell line (μg/mL)	287.16 ± 8.39 *	216.52 ± 11.90*
CC1 cell line(μg/mL)	555.03 ± 4.21*	842.01 ± 7.53*

*P < 0.01 when compared between AP (Aerial parts) and RP (Root parts)

The EC_50_ for MTT assay is significantly high (*P* < 0.001) for the RD cells treated with polyphenol free extracts compared to the crude extract (Figs. 9 & 10 and Table 4). Dose dependent increase in cytotoxicity was observed even in the absence of polyphenols at high concentrations. However the percentage cell viability in the absence of polyphenols was only 28.41 ± 2.94 and 26.77 ± 5.37 %, even at a concentration of 600 μg/mL with AP and RP respectively. At the same concentration AP and RP had 69.09 ± 0.70 and 63.36 ± 1.60 % cell viably respectively.

Hypophyllanthin and Phyllanthin are non polyphenolic compounds found in some *phyllanthus* species including *P. deblis* have shown that they exhibit anti-proliferative and antioxidant activity in vitro and *vivo* [38, 41]. The presence of non-phenolics which have anti-proliferative activity may have contributed to the cell death in the PVPP treated extracts (Fig. 10). However the contribution is low compared to the phenolic compounds.

The evidence supports that polyphenolic compounds are essentially responsible for cell death activating via the increase production of ROS (Reactive oxygen species). Based on the results it may be more effective to prescribe root instead of whole plant in therapy.

## Conclusions

The present study reveals that antioxidant activity and anti-proliferative activity are higher in root compared to aerial parts which associate with their phenolic content. Polyphenolic compounds present in both RP and AP extracts show a significant contribution for antioxidant activity and anti-proliferative activity against RD cell line. It was also found that *P. debilis* does not show cytotoxic activity against CC1 control cells as same as the cancer cells.

## Abbreviations

AAE: Ascorbic acid equivalent
AP: Aerial parts
CC1: normal rat liver cells
DPPH: 1,1-Diphenyl-2-picrylhydrazyl
EGCG: (−) -Epigallocatechingallate
GAE: Gallic acid equivalent
MTT: 3 4, 5-(dimethylthiazol-2-yl) 2-5-diphenyl tetrazolium bromide
PFAP: Polyphenol free aerial parts
PFRP: Polyphenol free root parts
PVPP: Polyvinyl polypyrrolidone
RD: Rhabdomyosarcoma cells
RP: Root parts
TPC: Total polyphenolic content
